# Radix Entomolaris: A Clinical Challenge

**DOI:** 10.5005/jp-journals-10005-1063

**Published:** 2010-08-17

**Authors:** Amit Kumar Garg, RK Tewari, MK Jindal, Neha Agrawal

**Affiliations:** 1 Lecturer, Department of Conservative Dentistry and Endodontics, Dr ZA Dental College, Aligarh Muslim University, Aligarh Uttar Pradesh, India; 2 Professor and Head, Department of Conservative Dentistry and Endodontics, Dr ZA Dental College, Aligarh Muslim University Aligarh, Uttar Pradesh, India; 3 Associate Professor and Chairman, Department of Pedodontics, Dr ZA Dental College, Aligarh Muslim University, Aligarh Uttar Pradesh, India; 4 Postgraduate Student, Department of Preventive and Community Dentistry, MS Ramaiah Dental College and Hospital, Bengaluru Karnataka, India

**Keywords:** Mandibular first molar, radix entomolaris, endodontic treatment, anatomical variation.

## Abstract

A major anatomical variant of the two-rooted mandibular first molar is a tooth with an additional distolingual third root: The radix entomolaris (RE). It is essential to anticipate and find all roots and canals during root canal treatment. Proper angulations and interpretation of radiographs help to identify pulp chamber and root anatomy. If present, an awareness and understanding of this unusual root and its root canal morphology can contribute to the successful outcome of root canal treatment.

## INTRODUCTION

Root canal anatomy and confounding nature of human pulpal system provide the majority of significant challenges in rendering endodontic treatment. Therefore, it is imperative that aberrant anatomy is identified prior to and during root canal treatment of such teeth.

Mandibular first molar can display several anatomical variations as the number of root canals and number of roots may also vary.^1^ The major variant in this tooth is the presence of an additional third root; a supernumerary root which can be found distolingually. This macrostructure, first mentioned in the literature by Carabelli, is called radix entomolaris (RE).^2^ The primary molars occasionally have an additional root located distolingually.

The incidence of a separate RE in the first mandibular molar is associated with certain ethnic groups as follows: European 3.4 to 4.2%, African 3%, Eurasian and Indian less than 5%,^3^ Caucasians 4.2%, Mongoloids such as Chinese, Eskimo and American Indians have 5% to more than 30% and the overall incidence in German dental school patient was 1.35%.^4^

## CASE REPORT

**Figs 1A to D:**
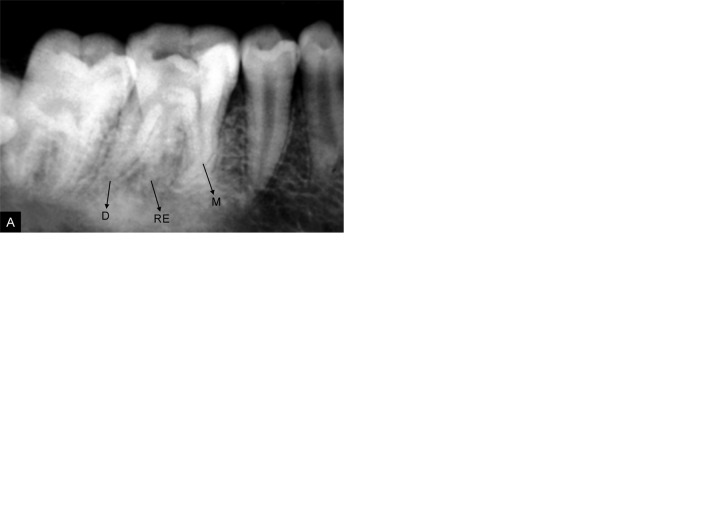
Radiograph reveals (A) An additional distolingual root (RE) (B) Working length determination (C) Master cone fit (D) Obturation (radix entomolaris in the middle)

A 16-year-old Indian female patient was referred for endodontic treatment of mandibular left first molar with chief complaint of spontaneous pain. The tooth was percussion sensitive, cold and heat sensitive, although there was no referred pain. Radiographical examination revealed an additional distolingual root (Fig. 1A). Tooth was anesthetized and isolated with rubber dam. The pulp chamber was opened, and one distal and two mesial canal orifices were located using an endodontic explorer (DG-16 Endodontic Explorer, Ash Instruments, Dentsply, Gloucester, United Kingdom). Upon visual inspection with a surgical loupe (Neitz BLS-3, Japan), a dark line was observed between the distal canal orifice and the distolingual corner of the pulp chamber floor. At this corner, overlying dentin was removed with a diamond bur with a noncutting tip (Diamendo, Dentsply Maillefer) and a second distal canal orifice was detected. The root canals were explored with a K-file #15 (Dentsply Maillefer, Ballaigues, Switzerland). The radiographical length measurement was performed with the Rinn set (Dentsply Rinn, Elgin, IL, USA) (Fig. 1B) and confirmed with an electronic apex locator (Raypex 5, VDW GmbH, Munchen, Germany). The root canals were shaped with protaper rotary instruments (Dentsply Maillefer) up to the F2-Protaper. During preparation EDTA (Glyde File Prep, Dentsply Maillefer) was used as a lubricant and the root canals were disinfected with a sodium hypochlorite solution (2.5%). The canals were obturated with protaper gutta-percha F2 (Figs 1C and D) and AH Plus sealer (Dentsply Maillefer). The pulp chamber was sealed with Ketac Fil glass ionomer cement (ESPE, Seefeld, Germany) and tooth was restored with silver amalgam.

## COMMENT

The tooth morphology of mandibular 1st molar may present ethnic variation, which can lead to treatment failure when not recognized. Every possible effort should be made towards locating of any extra root in mandibular 1st molars. Unusual tooth morphology, such as an extra cusp or a more prominent distal or distolingual lobe, in combination with a cervical convexity can indicate the presence of an additional root. All the clinical cases treated as third root present, unless otherwise, 30° mesial and distal radiographical shift and trapezoidal access opening excluded the presence of this macrostructure. When an additional root was present in a primary molar, the probability of the posterior adjacent molar also having an additional root was greater than 94.3%. The presence of an additional root in a primary molar can be used to predict the presence of an additional root in molars posterior to it.^5^
